# Characterization of the complete mitochondrial genome of Cassin’s auklet, *Ptychoramphus aleuticus*

**DOI:** 10.1080/23802359.2018.1495128

**Published:** 2018-08-13

**Authors:** Chang-Zhong Liu, Jin-Qing Jiang, Hui-Hui Zhang

**Affiliations:** College of Animal Science and Veterinary Medicine, Henan Institute of Science and Technology, Xinxiang, China

**Keywords:** Cassin’s auklet, *Ptychoramphus aleuticus*, mitochondrial genome, phylogenetic analysis

## Abstract

In this study, the complete mitochondrial genome of Cassin’s auklet, *Ptychoramphus aleuticus,* was determined using Illumina sequencing. The complete mitochondrial genome of *P. aleuticuss* was 16,524 bp in length and encoded 13 protein-coding genes, 22 transfer RNA (tRNA) genes, and 2 ribosomal RNA genes. The overall nucleotide composition is: 30.8% A, 24.4% T, 30.6% C, and 14.2% G, with a total G + C content of 44.8%. By phylogenetic analysis using ML method, *P. aleuticuss* clustered with two *Synthliboramphus* species that belong to Alcidae.

Cassin’s auklet (*Ptychoramphus aleuticus*), belonging to Alcidae, is a small, chunky seabird that ranges widely in the North Pacific. They are now at risk from a variety of factors, including oil spills and contaminants, introduced animals and plants, increased predation rates in response to artificial lights, and human disturbance (Shuford and Gardali 2008). And the population of *P. aleuticus* has apparently undergone a large and statistically significant decrease over the last 40 years in North America. Hence, *P. aleuticus* are evaluated and recognized as near threatened in the IUCN Red List.

In this study, the genomic DNA of *P. aleuticus* was extracted from muscle tissue of one adult specimen. The specimens were collected in Grayland Beach, Grays Harbor County, USA (46°80′N, 124°10′W). Both DNA and voucher specimen reside at the Zoology comprehensive laboratory from College of Animal Science and Veterinary Medicine. Library preparation was performed using the Illumina Nextera XT kit and whole genome sequencing was done on the Illumina Hiseq-2500 platform to produce 150 bp paired-end reads (BGI Tech, Shenzhen, China). After reads quality filtration, the clean reads were assembled by SPAdes 3.6.1 (Bankevich et al. 2012). Mitochondrial genome fragments were recycled using BLAST with other available Alcidae mitogenomes. To fill the gap, Price (Ruby et al. 2013) and MITObim version 1.8 (Hahn et al. 2013) were applied. The mitochondrial reads were collected by bowite2 (Langmead and Salzberg 2012) and reassembled. Then, Bandage (Wick 2015) was used to verify the circular structure of this mt genome. Thirteen PCGs and two rRNA genes were inferred based on comparison with mitogenome sequence of *Synthliboramphus antiquus* (Genbank accession: AP4009042.1) combined with predictions through the MITOS web server (Bernt et al. 2013) using the vertebrate mitochondrial genetic code. The location and secondary structure of the 22 tRNA were predicted using tRNAscan-SE Search Server version 1.21 (Lowe and Eddy 1997) and ARWEN (online version) (Laslett and Canback 2008).

The complete circular *Ptychoramphus aleuticus* mitogenome sequence (MH382811) is 16,524 bp in length and contains the typical metazoan set of 37 genes: 13 protein-coding genes, 22 tRNA and 2 rRNA genes, and an AT-rich control region. Among the 37 genes, there are 2 rRNAs, 12 PCGs, and 14 tRNAs encoded in the J strand, while ND6 and the rest 8 tRNAs are encoded in the N strand. The overall nucleotide composition is: 30.8% A, 24.4% T, 30.6% C, and 14.2% G, with a total G + C content of 44.8%.

To construct the phylogenetic relationship of *P. aleuticus* within Charadriiformes, other 29 complete mitogenomes derived from seven families were downloaded from GenBank. Among these mitogenomes, the distinct clade including Recurvirostridae, Haematopodidae, and Charadriidae was used as an outgroup. The genome-wide alignment of all mt genomes was done by HomBlocks (Bi et al. 2018), resulting in 12,488 positions in total, including almost all whole or partial PCGs and rRNA genes. These concatenated sets were used to reconstruct the phylogenetic relationships using Maximum Likelihood (ML) methods in MEGA6.0 (Tamura et al. 2013). The ML analysis was performed using default parameters and the confidence values of the ML tree were evaluated using a bootstrap test with 1000 iterations. As shown in Figure 1, the phylogenetic positions of these 30 mt genomes were successfully resolved with almost all bootstrap values beyond 90%. By phylogenetic analysis using ML method, *P. aleuticuss* clustered with two *Synthliboramphus* species that belong to Alcidae.

**Figure 1.**
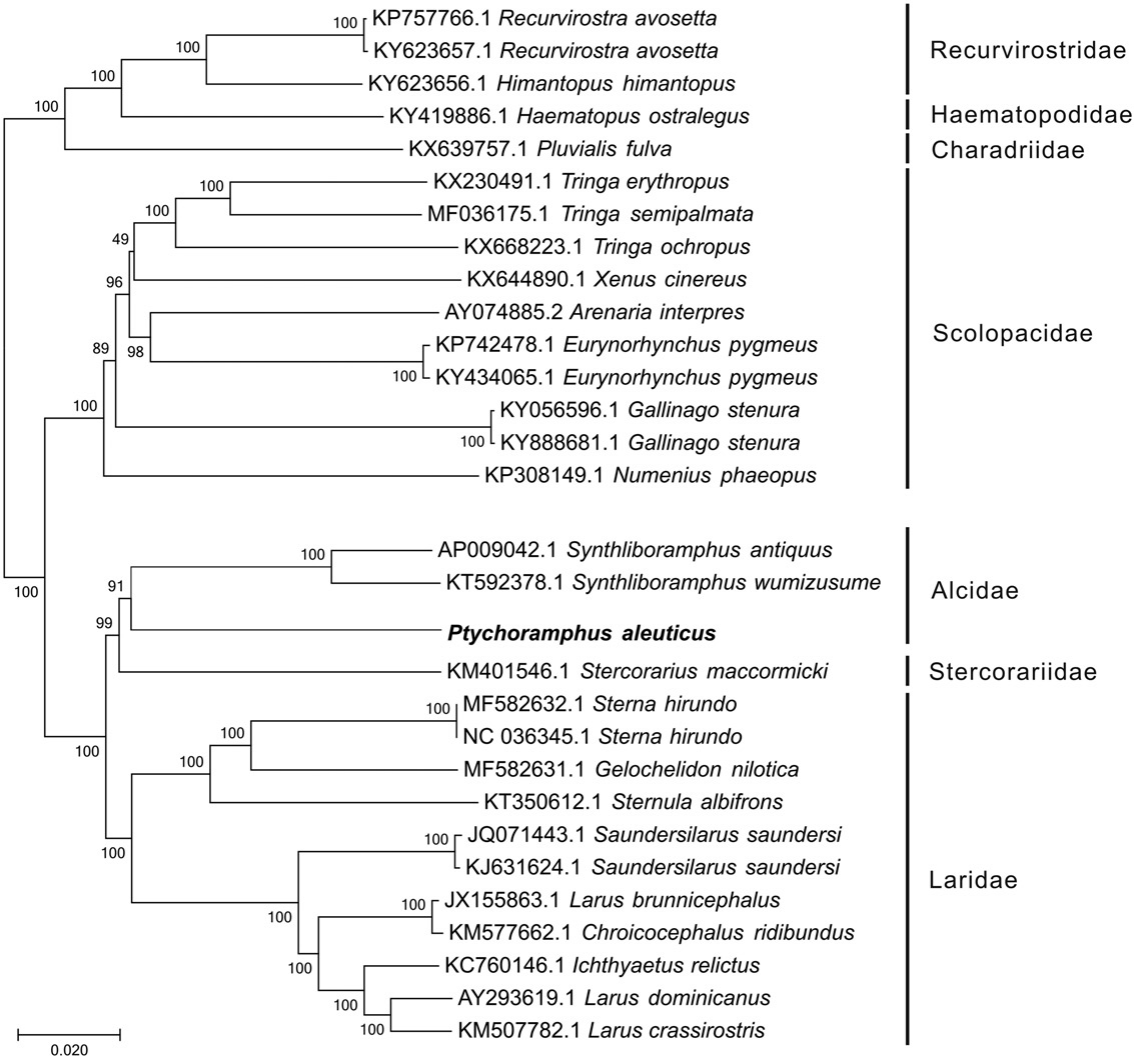
Phylogenetic relationships among 30 Charadriiformes mt genomes. Numbers beside divergence nodes are percents of 1000 bootstrap values. The length of branch represents the divergence distance.
